# Differences in neurosurgical treatment of intracerebral haemorrhage: a nation-wide observational study of 578 consecutive patients

**DOI:** 10.1007/s00701-019-03853-0

**Published:** 2019-03-15

**Authors:** Andreas Fahlström, Lovisa Tobieson, Henrietta Nittby Redebrandt, Hugo Zeberg, Jiri Bartek, Andreas Bartley, Maria Erkki, Amel Hessington, Ebba Troberg, Sadia Mirza, Parmenion P. Tsitsopoulos, Niklas Marklund

**Affiliations:** 1 Department of Neuroscience, Neurosurgery, Uppsala University, Uppsala University Hospital, SE-751 85 Uppsala, Sweden; 2 Department of Neurosurgery and Department of Clinical and Experimental Medicine, Linköping University, Linköping, Sweden; 3 Department of Clinical Sciences Lund, Neurosurgery, Lund University, Skane University Hospital, Lund, Sweden; 4 Department of Neuroscience, Karolinska Institutet, Stockholm, Sweden; 5 Department of Medicine and Clinical Neuroscience, Karolinska Institutet, Stockholm, Sweden; 6 Department of Neurosurgery, Karolinska University Hospital, Stockholm, Sweden; 7 Department of Neurosurgery, Copenhagen University Hospital Rigshospitalet, Copenhagen, Denmark; 8 Department of Clinical Neuroscience, Neurosurgery, University of Gothenburg, Sahlgrenska Academy, Sahlgrenska University Hospital, Gothenburg, Sweden; 9 Department of Clinical Neuroscience, Neurosurgery, Umeå University, Umeå University Hospital, Umeå, Sweden

**Keywords:** Intracerebral haemorrhage, Surgery, Guidelines, Craniotomy, External ventricular drain, Intraventricular haemorrhage

## Abstract

**Background:**

Supratentorial intracerebral haemorrhage (ICH) carries an excessive mortality and morbidity. Although surgical ICH treatment can be life-saving, the indications for surgery in larger cohorts of ICH patients are controversial and not well defined. We hypothesised that surgical indications vary substantially among neurosurgical centres in Sweden.

**Objective:**

In this nation-wide retrospective observational study, differences in treatment strategies among all neurosurgical departments in Sweden were evaluated.

**Methods:**

Patient records, neuroimaging and clinical outcome focused on 30-day mortality were collected on each operated ICH patient treated at any of the six neurosurgical centres in Sweden from 1 January 2011 to 31 December 2015.

**Results:**

In total, 578 consecutive surgically treated ICH patients were evaluated. There was a similar incidence of surgical treatment among different neurosurgical catchment areas. Patient selection for surgery was similar among the centres in terms of patient age, pre-operative level of consciousness and co-morbidities, but differed in ICH volume, proportion of deep-seated vs. lobar ICH and pre-operative signs of herniation (*p* < .05). Post-operative patient management strategies, including the use of ICP-monitoring, CSF-drainage and mechanical ventilation, varied among centres (*p* < .05). The 30-day mortality ranged between 10 and 28%.

**Conclusions:**

Although indications for surgical treatment of ICH in the six Swedish neurosurgical centres were homogenous with regard to age and pre-operative level of consciousness, important differences in ICH volume, proportion of deep-seated haemorrhages and pre-operative signs of herniation were observed, and there was a substantial variability in post-operative management. The present results reflect the need for refined evidence-based guidelines for surgical management of ICH.

## Introduction

Spontaneous supratentorial intracerebral haemorrhage (ICH) comprises approximately 10–20% of all stroke [10], with an annual incidence of around 20 cases per 100,000 [2, 4]. Mortality rate reaches 40% at 1 month, 54% at 1 year and only 12–39% of the survivors reach functional independence [4]. Negative prognostic factors include large ICH volume, non-lobar location, intraventricular extension of haemorrhage, poor neurological status and high age [3, 18, 35, 42].

Treatment for ICH encompasses both medical and surgical strategies [8, 50, 51]. The rationale for surgical intervention is reduction of mass effect and elevated intracranial pressure (ICP), as well as a possible improvement in perihaemorrhagic brain tissue environment by enhancing cerebral perfusion and by removing neurotoxic substances [24, 29, 34, 45, 48]. Whether or not surgery improves the outcome of patients with spontaneous supratentorial ICH and is superior to best medical treatment is a subject of intense debate and controversy [11]. There are a number of small [6, 7, 15, 21, 33, 37, 44, 52] and two large [30, 32] randomised controlled trials (RCT) of surgically treated ICH patients showing varying mortality and morbidity. Neither of the two large RCTs conducted in recent years, STICH [30] and STICH II [32], showed significant differences between early surgery versus best medical treatment, although patient inclusion criteria and large cross-over between treatment groups limit the generalizability of the results. Additionally, two meta-analyses of such RCTs [14, 39] both found a small but significant benefit of surgical treatment over best medical management, albeit with large data heterogeneity. Guidelines defining indications for surgical treatment are still lacking [18, 42] which plausibly opens up for large variation in the treatment provided to ICH patients [13]. Moreover, to date, treatment differences among neurosurgical centres have not been thoroughly evaluated [11].

There are national and regional stroke guidelines in Sweden [41], in line with the American and European guidelines [18, 42]. However, these address mainly the medical management of ICH, whereas the indications for neurosurgical treatment and post-operative ICH treatment are not outlined in detail, and are thus open for individual interpretation and potentially biased decision making [9].

Sweden has a tax-funded homogenous health care system, which ensures the population equal access to health care services. Furthermore, neurosurgical care is only provided by six centres in Sweden, allocated in different tertiary referral hospitals. They each serve a defined geographic area, and together, they are responsible for all neurosurgical care of the 10 million inhabitants.

We performed a nation-wide retrospective observational study of all neurosurgically treated patients with ICH in Sweden during a 5-year period (2011–2015). The main aim of this study was to systematically elucidate similarities and differences in treatment and indication for surgery among neurosurgical centres in Sweden, in ICH patients, for whom defined clinical guidelines are lacking.

## Method

Data was collected by examining medical records and neuroimaging from all six neurosurgical centres in Sweden (Lund, Gothenburg, Linköping, Stockholm, Uppsala and Umeå) during the period 1 January 2011 to 31 December 2015. The study was approved by the regional board of ethical review in Uppsala, Sweden.

The neurosurgical centres are found in the following hospitals (from south to north) in Sweden with their respective population catchment size indicated in parenthesis: Lund (1.8 million), Gothenburg (1.8 million), Linköping (1.0 million), Stockholm (2.3 million), Uppsala (2.0 million) and Umeå (0.9 million).

Patients with a first-listed ICD-10 diagnosis code I61.0-9 admitted and treated during the 5-year period were evaluated for inclusion. Those included were adult patients with neurosurgically treated supratentorial intracerebral haemorrhage (ICH), while patients with infratentorial ICH (cerebellum and brainstem), and ICH related to trauma, neoplasm, vascular malformation, venous sinus thrombosis or a haemorrhagic transformation of ischemic stroke were excluded, as were patients not undergoing surgery.

Patients’ age, gender and past medical history including hypertension, diabetes, previous myocardial infarction and previous stroke was documented. Treatment with antihypertensive-, antiplatelet-, warfarin and non-vitamin K oral anticoagulant drugs (NOACs) was recorded as well as information on neuroimaging with computed tomography scan (CT), CT angiography (CTA), magnetic resonance imaging (MRI) and digital subtraction angiography (DSA) performed during the acute treatment period. Based on the CT imaging, the hematoma was defined as either lobar or deep-seated. Lobar hematoma incorporated haemorrhages in the frontal, parietal, temporal and occipital lobes originating from the cortex and subcortical white matter [19, 31]. Deep-seated hematomas comprised haemorrhages originating from the basal ganglia and thalamus [19, 30, 46]. The presence of hydrocephalus (HC) [47] and intraventricular haemorrhage (IVH) was documented. The severity of IVH was graded using the LeRoux numerical scoring system [28]. The volume of the intracerebral hematoma was calculated using the ABC/2 technique [27, 49]. Glasgow coma scale motor response (GCS-M), pupillary size, asymmetry and reaction to light, arm and leg paresis/paralysis and dysphasia were documented preoperatively and at discharge, as was the length of stay (LOS) in the neurocritical care (NCC) unit. A possible transtentorial herniation was defined as a decreased level of consciousness in combination with signs indicating compression of the third cranial nerve such as uni- or bilateral pupil dilatation with or without sluggish or unresponsive reaction to light.

The type of surgical procedure, craniotomy and ICH evacuation, treatment with EVD alone, or other surgical technique was documented and the number of reoperations was noted. The use of ICP monitoring and the number of days with ICP monitoring were recorded, as was the use and duration of any drainage of cerebrospinal fluid (CSF) and mechanical ventilator. Primary end-point was 30-day mortality.

The total incidence of supratentorial intracerebral haemorrhage in our country was retrieved from the National Stroke Register, which has a 90% national coverage [36].

Statistical analysis was done with SPSS Statistics 22. Continuous variables were presented as mean (SD) or median (IQR 25th–75th percentile) values. Categorical variables were presented as numbers and percentages. Chi-square test was used for comparison of proportions of categorical variables. Adjusted residuals were investigated to localise effect as sample sizes were uneven among groups, and >± 1.96 (2SD) was considered indicative of effect location. Continuous variables were analysed with ANOVA, and multiple comparisons using Games-Howell were used for post hoc analysis. Missing values were excluded from analysis. Patients lost to follow-up (*N* = 4) were excluded from analysis. All tests were two-tailed. A *p* value of < .05 was considered statistically significant.

## Results

Between 1 January 2011 and 31 December 2015, 578 patients (mean age 59 ± 12 years) were treated surgically for supratentorial ICH in Sweden. Of these, 39% were female and 61% were male, with 402 patients treated with craniotomy and ICH evacuation, whereas 176 were treated with EVD alone. The incidence of neurosurgical intervention was 1.37/100,000 inhabitants for all included patients, 0.8/100,000 for those treated with craniotomy and ICH evacuation and 0.4/100,000 for those treated by EVD only (Table 1). The incidence of supratentorial ICH (approximately 80% of all ICH) in Sweden in the study time period was approximately 2370 patients per year [36]. Thus, approximately 5% of all patients with supratentorial ICH in the country were treated surgically during the study period, with similar incidence of neurosurgical intervention among the different centres (Table 1).

**Table 1:** Incidence of surgical intervention for intracerebral haemorrhage per 100,000 inhabitants, presented for each centre

Incidence per 100,000 inhabitants							
	Lund	Gothenburg	Linköping	Stockholm	Uppsala	Umeå	All
Entire patient cohort	1.26	0.75	1.53	1.03	1.22	1.72	1.17
Craniotomy	1.03	0.47	1.07	0.65	0.79	1.31	0.82
EVD alone	0.22	0.28	0.47	0.38	0.43	0.41	0.36

*EVD* external ventricular drainage

**Fig. 1:**
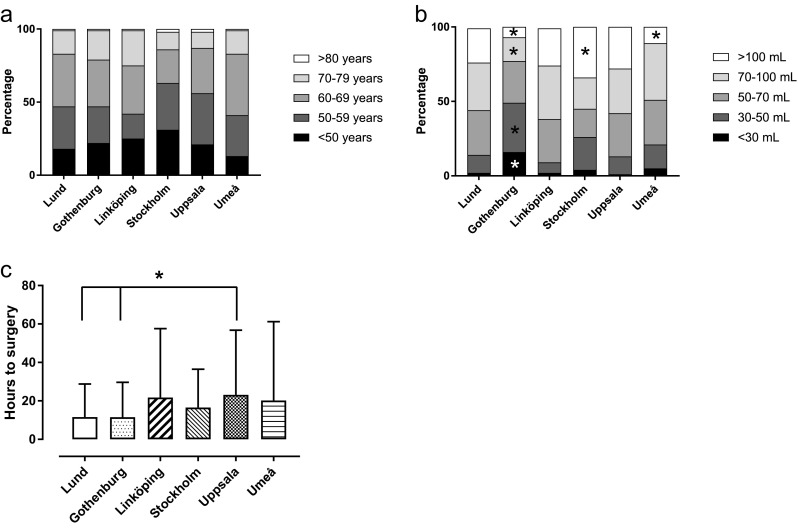
**a** Age distribution of patients at the centres showing percentage of all patients in each category divided by decade from 50 to 80. There was no significant difference in the age of the patients treated surgically (*χ*^2^(20,577) = 27.38, *p* = .13). **b** Variations in hematoma volume of patients treated by craniotomy and ICH evacuation among the different centres. *Significantly different proportion compared to other centres (*χ*^2^(5) = 52.79, *p* < .001). **c** Difference in time to surgery (hours) from ICH onset. *A significant difference between the centres (*F*(5) = 2.8, *p* = .02)

The average patient age did not differ significantly between centres (*χ*^2^(20,577) = 27.4, *p* = .125; Fig. 1a; grouped by < 50 years, then each decade until 80 years), nor did the preoperative GCS-M score grouped into 1–2, 3–4 and 5–6, respectively (*χ*^2^(10,545) = 13.13, *p* = .22; Table 2). Patient age did not differ between centres when analysed also as a continuous variable (*F*(5) = 1.6, *p* = .16). The proportion of patients with signs of possible transtentorial herniation was significantly higher at one centre (Umeå) compared to the others (*χ*^2^(5) = 14.5, *p* = .01; Table 2). There was a difference among centres in the proportion of females in the entire cohort (*χ*^2^(5) = 13.5, *p* = .019; Table 2). The proportion of left-sided ICH in the entire patient cohort differed among centres (*χ*^2^(5) = 13.8, *p* = .017; Table 2).

**Table 2:** Patient characteristics. *N*(%)

Patient characteristics entire cohort, *N*(%)								
		Lund *N* = 112	Gothenburg *N* = 69	Linköping *N* = 79	Stockholm *N* = 118	Uppsala *N* = 124	Umeå *N* = 76	*p* value
Female		40(36)	36(52)*	36(46)	44(37)	50(40)	*19(25)‡*	.019
GCS-M on admission								.217
	1–2	4(4)	4(6)	9(12)	7(7)	4(3)	5(7)	
	3–4	17(15)	18(26)	15(20)	16(17)	19(16)	11(15)	
	5–6	90(81)	47(68)	52(68)	71(76)	99(81)	57(78)	
Abn. Pupil(s)		28(25)	*7(10)‡*	16(20)	20(17)	16(13)	22(29)*	.013
Left hemisphere		47(42)	26(38)	43(54)	51(43)	74(60)*	35(46)	.017
HC		*33(30)‡*	24(35)	37(47)	68(58)*	74(60)*	*16(21)‡*	< .001
IVH		61(61)	45(65)	60(77)	81(69)	91(75)	57(75)	.113
Craniotomy		*92(82)‡*	43(62)	55(70)	74(63)	80(65)	58(76)	.007
Deep-seated		60(54)	41(59)	60(76)*	*49(42)‡*	84(68)*	47(62)	< .001

*Significantly higher than other centres

‡Significantly lower than other centres. *P* < .05 is indicated by italics

*N* number of patients, *GCS-M* Glasgow Coma Score Motor component, *Abn. Pupil(s)* one or two dilated pupils with abnormal reaction to light, *HC* hydrocephalus, *IVH* intraventricular haemorrhage

The average hematoma volume was significantly smaller at one centre (Gothenburg) compared to all others (*χ*^2^(5) = 52.8, *p* < .001; Fig. 1b; grouped as < 30 mL; 30–50 mL; 50–70 mL; 70–100 mL; > 100 mL). This difference remained statistically significant also when volume was analysed as a continuous variable (*F*(5) = 5.8, *p* < .001). Time to surgery varied between centres (*F*(5) = 2.8, *p* = .02; Fig. 1c).

The prevalence of co-morbidities, including previously known hypertension, acute untreated hypertension, diabetes, previous myocardial infarction, antihypertensive drugs, antiplatelet drugs, warfarin and non-vitamin K anticoagulant (NOAC) drugs, was similar across centres, although one centre had a higher frequency of patients with prior stroke (*χ*^2^(5) = 18.2, *p* = .003; Table 3).

**Table 3:** Frequency of co-morbidities of entire patient cohort. *N*(%)

Comorbidities, *N*(%)							
	Lund	Gothenburg	Linköping	Stockholm	Uppsala	Umeå	*p value*
VKA	12 (11)	4 (6)	12 (15)	6 (5)	15 (12)	10 (13)	.160
NOAC	1 (1)	0 (0)	0 (0)	1 (1)	4 (3)	0 (0)	–
Antiplatelet	17 (15)	14 (20)	16 (20)	17 (15)	22 (18)	13 (17)	.876
Thrombolytic	2 (2)	0 (0)	3 (4)	0 (0)	1 (1)	0 (0)	–
DM Type I	1 (1)	5 (7)	2 (3)	1 (1)	3 (2)	1 (1)	.071
DM Type II	13 (12)	9 (13)	11 (14)	15 (13)	12 (10)	9 (12)	.956
HT (med)	44 (39)	36 (52)	35 (44)	52 (44)	70 (57)	41 (54)	.085
HT (no med.)	41 (37)	32 (46)	25 (32)	40 (34)	51 (41)	34 (45)	.300
Previous MI	8 (7)	8 (12)	7 (9)	8 (7)	7 (6)	6 (8)	.769
Previous CVL	11 (10)	7 (10)	7 (9)	11 (9)	29 (23)*	6 (8)	.003

*Significantly higher than other centres

*P* < .05 is indicated by italics

*VKA* vitamin-K antagonist, *NOAC* non vitamin-K oral anticoagulant, *DM* diabetes mellitus, *HT* hypertension, *med* medicated, *no med* not on medication, *MI* myocardial infarction, *CVL* cerebrovascular lesion

In the entire cohort, the choice between craniotomy and ICH evacuation versus treatment with EVD alone differed significantly among centres (*χ*^2^(5) = 15.8, *p* = .007; Table 2). The frequency of craniotomy and ICH evacuation was similar for lobar haemorrhages (91–100%), although differed significantly (*p* < .001) for deep-seated haemorrhages, ranging from 22 to 68% treated by craniotomy and ICH evacuation (Table 4), while the remaining were treated by EVD only.

**Table 4:** Surgical method in deep-seated vs lobar ICH. *N*(%)

Proportion undergoing craniotomy, *N*(%)							
	Lund *N* = 112	Gothenburg *N* = 69	Linköping *N* = 79	Stockholm *N* = 118	Uppsala *N* = 124	Umeå *N* = 76	*p* value
Entire patient cohort	92(82)*	43(62)	55(70)	74(63)	80(65)	58(76)	.007
	Lund *N* = 60	Gothenburg *N* = 41	Linköping N = 60	Stockholm *N* = 49	Uppsala *N* = 84	Umeå *N* = 47	*p* value
Deep-seated ICH	41(68)*	*15(37)‡*	36(60)	*11(22)‡*	42(50)	30(64)	< .001
	Lund *N* = 52	Gothenburg *N* = 28	Linköping *N* = 19	Stockholm N = 69	Uppsala *N* = 40	Umeå *N* = 29	*p* value
Lobar ICH	51(98)	28(100)	19(100)	63(91)	38(95)	28(97)	.281

*Significantly higher than other centres

‡Significantly lower than other centres. *P* < .05 is indicated by italics

*N* number of patients, *ICH* intracerebral haemorrhage

In addition, the proportion of deep-seated hematomas compared to lobar hematomas undergoing any surgical treatment differed between centres with two centres (Linköping and Uppsala) operating significantly more deep-seated ICH and another centre (Stockholm) significantly more lobar ICH (*χ*^2^(5) = 29.8, *p* < .001; Table 4). There was a small number of patients (*n* = 9, 1.6%) in the entire patient cohort undergoing surgery classed as “other” which included endoscopic surgery. The frequency of reoperations was 8% in the entire cohort, and this did not differ significantly between centres (*χ*^2^(5) = 10.2, *p* = .07).

In the group of patients treated by craniotomy and ICH evacuation, there was no difference between centres in the number of patients with intraventricular haemorrhage (*χ*^2^(5) = 8.9, *p* = .064), whereas the frequency of hydrocephalus differed among centres (*χ*^2^(5) = 48.8, *p* < .001; Table 5), as it did also for the entire patient cohort (*χ*^2^(5) = 49.8, *p* < .001; Table 2).

**Table 5:** ICH characteristics divided by treatment choice. *N*(%)

ICH characteristics, *N*(%)							
Patients treated with craniotomy with ICH evacuation							
	Lund *N* = 90	Gothenburg *N* = 43	Linköping *N* = 55	Stockholm *N* = 74	Uppsala *N* = 80	Umeå *N* = 58	*p* value
IVH	43(52)	19(44)	37(69)	39(53)	50(63)	39(67)	.064
HC	*16(17)‡*	*1(2)‡*	15(27)	30(41)*	36(45)*	*5(9)‡*	< .001
Left hemisphere	39(42)	15(35)	28(51)	28(38)	44(55)	28(48)	.167
Deep-seated	41(45)	15(35)	36(66)*	*11(15)‡*	42(53)	30(52)	< .001
Female	35(38)	21(49)	22(40)	27(37)	29(36)	12(21)	.091
Patients treated with EVD alone							
	Lund *N* = 20	Gothenburg *N* = 26	Linköping *N* = 24	Stockholm *N* = 44	Uppsala *N* = 44	Umeå *N* = 18	*p* value
IVH	18(100)	26(100)	23(96)	42(96)	41(98)	18(100)	.732
HC	17(85)	23(89)	22(92)	38(86)	38(86)	11(61)	.106
Left hemisphere	6(30)	11(42)	7(29)	23(52)	24(55)	7(39)	.210
Deep-seated	19(95)	26(100)	24(100)	38(86)	42(96)	17(94)	.139
Female	5(25)	15(58)	14(58)	17(39)	21(48)	7(39)	.169

*Significantly higher than other centres

‡Significantly lower than other centres. *P* < .05 is indicated by italics

*N* number of patients, *ICH* intracerebral haemorrhage, *IVH* intraventricular haemorrhage, *HC* hydrocephalus, *EVD* external ventricular drain

All centres performed a preoperative computed tomography (CT). The use of CT-angiography varied between 39.1 and 76.3% between centres (*χ*^2^(5) = 40.0, *p* < .001; Table 6). Additional neuroimaging was performed in several centres including magnetic resonance imaging, contrast-enhanced CT and digital subtraction angiography (Table 6).

**Table 6:** Neuroimaging used during the treatment period. *N*(%)

Neuroimaging, *N*(%)							
	Lund	Gothenburg	Linköping	Stockholm	Uppsala	Umeå	*p value*
CT	109 (97)	69 (100)	79 (100)	117 (99)	121 (98)	76 (100)	.27
CTA	57 (51)	27 (39)	46 (58)	90 (76)*	53 (43)	49 (65)	.000
Other§	9 (8)	2 (3)	10 (13)	15 (13)*	4 (3)	2 (3)	.007
§ *Other imaging techniques¥:*							
*CECT*	*6 (5)*	*2 (3)*	*2 (3)*	*5 (4)*	*1 (1)*	*0 (0)*	*–*
*MRI*	*2 (2)*	*0 (0)*	*7 (9)*	*9 (8)*	*2 (2)*	*2 (3)*	*–*
*MRA*	*0 (0)*	*0 (0)*	*0 (0)*	*0 (0)*	*1 (1)*	*0 (0)*	*–*
*DSA*	*2 (2)*	*0 (0)*	*1 (1)*	*4 (3)*	*1 (1)*	*0 (0)*	*–*

*Significantly higher than other centres

‡Significantly lower than other centres. *P* < .05 is indicated by italics. § indicates other neuroimaging. ¥-numbers too small for statistical analysis

*N* number of patients, *CT* computer tomography, *CTA* computer tomography angiography, *Other* all other neuroimaging, including: *CECT* contrast-enhanced computer tomography, *MRI* magnetic resonance imaging, *MRA* magnetic resonance imaging angiography, *DSA* digital subtraction angiography

**Fig. 2:**
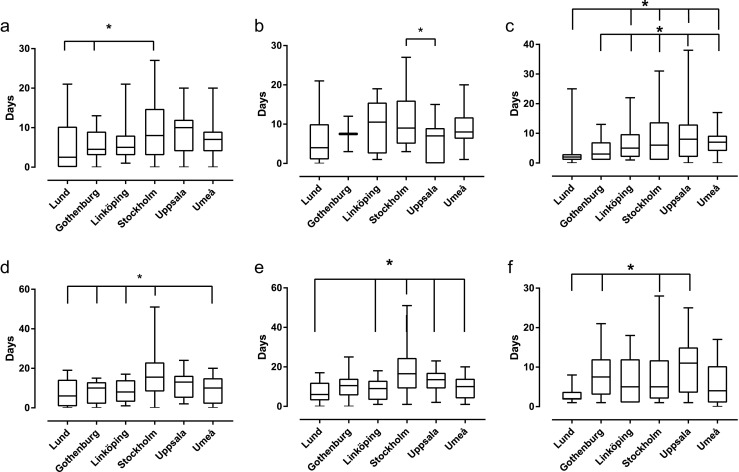
For patients treated with craniotomy with ICH evacuation, the duration of use of **a** ICP-monitoring, **b** CSF-drainage and **c** mechanical ventilation differed significantly between centres as it did for patients treated by EVD alone where ICP-monitoring, CSF-drainage and mechanical ventilation are shown in **d**, **e** and **f**, respectively. Abbreviations: ICH = intracerebral haemorrhage; EVD = external ventricular drainage; ICP = intracranial pressure; CSF = cerebrospinal fluid. *p < .05

Post-operative management showed some variability among centres. In patients treated with craniotomy, two centres used less ICP monitoring than the others (χ^2^ (5) = 112.1, *p* < .001; Table 7) and the duration of ICP monitoring differed (*F*(5) = 4.0, *p* = .002; Fig. 2a), as did the duration of CSF drainage (*F*(5) = 3.1, *p* = .01; Fig. 2b). The use of mechanical ventilation also varied significantly in frequency (*χ*^2^(5) = 21.1, *p* = .001; Table 7) and duration (*F*(5) = 8.3, *p* < .001; Fig. 2c). For patients treated with EVD alone, the use of ICP-monitoring (*χ*^2^(5) = 63.6, *p* < .001) and CSF drainage (*χ*^2^(5) = 36.3, *p* < .001) differed among centres (Table 7) as did the duration of ICP-monitoring (*F*(5) = 6.6, *p* < .001), CSF-drainage (*F*(5) = 7.9, *p* < .001) and mechanical ventilation (*F*(5) = 4.0, *p* = .002; Fig. 2d–f).

**Table 7:** Neurocritical care parameters. *N*(%)

Neurocritical care strategies, *N*(%)							
Patients treated by craniotomy and ICH evacuation							
	Lund *N* = 90	Gothenburg *N* = 43	Linköping *N* = 55	Stockholm *N* = 74	Uppsala *N* = 80	Umeå *N* = 58	*p* value
ICP-monitoring	*26(28)‡*	*12(28)‡*	45(82)*	40(54)	67(84)*	53(91)*	< .001
CSF-drainage	*15(16)‡*	*2(5)‡*	12(22)	29(39)*	31(39)*	20(35)	< .001
Mechanical ventilation	*60(66)‡*	31(72)	48(87)	56(77)	67(84)	54(93)*	.001
Patients treated by EVD alone							
	Lund *N* = 20	Gothenburg *N* = 26	Linköping *N* = 24	Stockholm *N* = 44	Uppsala *N* = 44	Umeå *N* = 18	*p* value
ICP-monitoring	17(85)	*11(42)‡*	23(96)	43(98)*	43(98)*	18(100)	< .001
CSF-drainage	16(80)	24(92)	20(83)	43(98)*	*24(55)‡*	18(100)*	< .001
Mechanical ventilation	12(60)	22(85)	19(79)	36(82)	37(84)	17(94)	.131

*Significantly higher than other centres

‡Significantly lower than other centres. *P* < .05 is indicated by italics

*N* number of patients, *ICH* intracerebral haemorrhage, *ICP* intracranial pressure, *CSF* cerebrospinal fluid, *EVD* external ventricular drain

One centre (Stockholm) was unable to provide detailed data on length of stay (LOS) in a NCC unit. The mean LOS in the NCC unit of the remaining centres was 7.7 (± 6) days, with significant differences among centres (data not shown; *F*(4) = 7.4, *p* < .001). The organisation of neurocritical care differed between centres with five having dedicated neurocritical care units (Lund, Gothenburg, Linköping, Stockholm and Uppsala), whereas one (Umeå) treated neurocritical care patients within the general ICU setting.

The 30-day mortality was similar between centres for patients treated by EVD alone but showed a small but significant difference for patients treated by craniotomy and ICH evacuation (*χ*^2^(5) = 17.1, *p* = .026; Table 8) and for the entire patient cohort (*p* = .004; Table 8). The 30-day mortality for the entire cohort grouped by preoperative GCS-M score was 50% (*n* = 4/8) for GCS-M 1, 42% (*n* = 10/24) for GCS-M 2, 21% (*n* = 6/28) for GCS-M 3, 25% (*n* = 17/68) for GCS-M 4, 16% (*n* = 35/215) for GCS-M 5 and 10% (*n* = 20/198) for GCS-M 6. Overall 30-day mortality was 17% (*n* = 96/574).

**Table 8:** 30-day mortality. *N*(%)

30-day mortality, *N*(%)							
	Lund	Gothenburg	Linköping	Stockholm	Uppsala	Umeå	*p* value
Entire patient cohort	*11(10)‡*	10(15)	20(25)*	13(11)	21(17)	21(28)*	.004
Craniotomy	9(10)	4(9)	13(24)*	5(7)	12(15)	13(22)*	.026
EVD	2(10)	6(23)	7(29)	8(19)	9(21)	8(44)	.170

*Significantly higher than other centres

‡Significantly lower than other centres. *P* < .05 is indicated by italics

*N* number of patients, *EVD* external ventricular drain

## Discussion

This is the first study evaluating nation-wide differences in surgical management of intracerebral haemorrhage (ICH). We compared the surgical indications, treatment and post-operative management of patients with supratentorial ICH among the neurosurgical centres in Sweden during a 5-year period. In previous studies evaluating trends in surgical management of ICH, either in samples of the population or in single centres, a large variability in treatment strategies has been found [1, 5, 25]. A subgroup analysis of the STICH-trial data suggested large international variations in surgical practice and treatment of ICH [13], highlighting the need for studies of large cohorts of ICH patients to characterise the variability of management and its impact on outcome. A recent retrospective single-centre study showed favourable outcome following surgery for ICH, similar for both deep-seated and lobar location, thus adding to the ongoing debate on the indications for surgical intervention in ICH [19].

Our study shows that the frequency of surgical treatment of patients with supratentorial ICH in Sweden was 5%, which is in line with previous studies [1, 13, 25, 38]. The number of patients treated at the different neurosurgical centres was similar when adjusted for the size of their respective catchment populations. The majority of patients were treated with craniotomy and ICH evacuation. Patient’s age and medical history, preoperative neurological status, presence of intraventricular blood and/or hydrocephalus were comparable among the six centres indicating, to some degree, similar criteria for neurosurgical intervention in our country. In addition, the number of reoperations was similar. However, ICH volume, the proportion of deep-seated haemorrhages, use of ICP-monitoring, CSF drainage, post-operative mechanical ventilation and length of stay in neurocritical care unit showed variability between centres. Importantly, although mortality rates were slightly higher in some centres, we cannot conclude that the observed management differences influenced mortality.

The subgroup analysis of the STICH trial suggested a tentative benefit of early surgery over conservative treatment for patients with an ICH < 1 cm from the cortical surface [30], i.e. a lobar location of ICH. These observations led to protocol used in the subsequent STICH II trial. In our present study, the difference in mortality rates for patients treated by craniotomy might reflect the variation in the proportion of deep-seated ICH evacuated by craniotomy. The 30-day mortality in our large ICH cohort was nevertheless comparable to, or lower than, mortality reported in previous studies of surgically treated ICH patients [1, 5, 13, 30, 38]. Furthermore, 30-day mortality rates in this study are lower than those predicted for the entire ICH patient population [17, 40], possibly suggesting a benefit of surgical treatment. European Guidelines for ICH treatment state that patients presenting with GCS 9-12 may have the best clinical benefit of surgery [42]. In the present cohort, the 30-day mortality for the poor-grade patients (GCS-M 1-3) was as low as 21–50%, arguing for a lifesaving role of ICH surgery in combination with neurocritical care. This can be contrasted to the STICH cohort where a uniformly bad outcome was reported in comatose patients [30].

In the present study, only 1.6% of patients were treated with surgical methods other than craniotomy and ICH evacuation or EVD treatment alone, including endoscopic surgery. Our data show that minimally invasive surgery (MIS) was not often used in Sweden in 2011–2015; however, with accumulating research data on MIS becoming gradually available, this might change in the future, provided that a clinical benefit of MIS is shown.

Time to surgery differed significantly between centres, likely reflecting shorter distances from referral hospitals to the neurosurgical centres in some areas of our country. This may explain why the northernmost neurosurgical department (Umeå), covering a large geographic area [43], had significantly more patients showing signs of possible transtentorial herniation prior to surgery.

Neurocritical care is organised differently at the various centres where some use dedicated neurocritical care units whereas others use a general ICU. This could contribute to the differences in neurocritical care management noted in this study, including the use of ICP monitoring, and early extubation.

Limitations of the present study include the lack of long-term follow-up, detailed functional outcome and quality-of-life data as well as the retrospective study design. The majority of ICH studies report outcome up to 3 or 6 months post ICH onset and only a few studies report on long-term outcome after ICH [12, 26]. Determining a long-term prognosis for functional outcome in the acute phase is difficult for ICH patients, further complicated by a shortage of long-term outcome studies [23], thus a long-term follow-up of the present cohort is warranted.

Another limitation of the study is the lack of details on medical management, such as specific systolic blood pressure levels or choice of antihypertensive medication, which might have added valuable information. Presumably, all centres aim for blood pressure control in accordance with current guideline recommendations, thus limiting variability in these parameters, although our pragmatic study design does not allow us to confirm this.

A strength of this present study is the comprehensive, nation-wide inclusion of each patient subjected to surgical treatment for ICH, which, to our knowledge, has not been done previously. The multi-centre design of this study enabled the detection of both differences and similarities in ICH management, highlighting the need for refined guidelines. In many aspects, the criteria for neurosurgical intervention in Sweden were homogenous across centres. This could, in part, be explained by a uniform resident training through the Nordic (www.neurosurgery.no), and the European Association of Neurosurgical Societies (EANS) training courses, attended by the majority of neurosurgical residents in Sweden. However, in view of the lack of detailed management protocols, local treatment policies may develop into different treatment and management strategies which could markedly influence patient outcome [16, 20, 22, 23].

## Conclusion

Our study, the first nation-wide study of neurosurgically treated ICH patients, shows that a congruent health care organisation and a rather homogeneous training of neurosurgeons can result in similarities in selection criteria for neurosurgical intervention. Nevertheless, there were still important differences in patient selection, choice of surgical method, and post-operative management among neurosurgical centres, plausibly reflecting regional practices and more importantly a lack of defined guidelines for many aspects of ICH care. Our results identify areas where additional studies are needed to provide better evidence for ICH management. Future studies should aim to determine guidelines for postoperative care of surgically treated ICH patients and should also characterise the long-term functional outcome and quality of life in large cohorts of surgically treated ICH-patients.

## Abbreviations

EVD: external ventricular drain
ICH: intracerebral haemorrhage
CSF: cerebrospinal fluid
ICP: intracranial pressure
RCT: randomised controlled trial
CT: computed tomography
ICD-10: International Statistical Classification of Diseases and Related Health Problems - Tenth Revision
MRI: magnetic resonance imaging
DSA: digital subtraction angiography
HC: hydrocephalus
IVH: intraventricular haemorrhage
GCS-M: Glasgow Coma Scale Motor score
LOS: length of stay
NCC: neurocritical care
NOAC: non-vitamin K anticoagulant drug
